# A Comparative Review of microRNA Expression Patterns in Autism Spectrum Disorder

**DOI:** 10.3389/fpsyt.2016.00176

**Published:** 2016-11-04

**Authors:** Steven D. Hicks, Frank A. Middleton

**Affiliations:** ^1^Department of Pediatrics, Penn State College of Medicine, Hershey, PA, USA; ^2^Department of Neuroscience and Physiology, SUNY Upstate Medical University, Syracuse, NY, USA; ^3^Department of Psychiatry and Behavioral Sciences, SUNY Upstate Medical University, Syracuse, NY, USA; ^4^Department of Biochemistry and Molecular Biology, SUNY Upstate Medical University, Syracuse, NY, USA

**Keywords:** autism, microRNA, neurodevelopment, biomarker

## Abstract

Autism spectrum disorder (ASD) is a neurodevelopmental disorder characterized by a wide spectrum of deficits in social interaction, communication, and behavior. There is a significant genetic component to ASD, yet no single gene variant accounts for >1% of incidence. Posttranscriptional mechanisms such as microRNAs (miRNAs) regulate gene expression without altering the genetic code. They are abundant in the developing brain and are dysregulated in children with ASD. Patterns of miRNA expression are altered in the brain, blood, saliva, and olfactory precursor cells of ASD subjects. The ability of miRNAs to regulate broad molecular pathways in response to environmental stimuli makes them an intriguing player in ASD, a disorder characterized by genetic predisposition with ill-defined environmental triggers. In addition, the availability and extracellular stability of miRNAs make them an ideal candidate for biomarker discovery. Here, we discuss 27 miRNAs with overlap across ASD studies, including 3 miRNAs identified in 3 or more studies (miR-23a, miR-146a, and miR-106b). Together, these 27 miRNAs have 1245 high-confidence mRNA targets, a significant number of which are expressed in the brain. Furthermore, these mRNA targets demonstrate over-representation of autism-related genes with enrichment of neurotrophic signaling molecules. Brain-derived neurotrophic factor, a molecule involved in hippocampal neurogenesis and altered in ASD, is targeted by 6 of the 27 miRNAs of interest. This neurotrophic pathway represents one intriguing mechanism by which perturbations in miRNA signaling might influence central nervous system development in children with ASD.

## Background

In 1993, the first non-coding antisense RNA sequence was described in *Caenorhabditis elegans* (1, 2) and termed microRNA (miRNA). Over the next 10 years, the roles of miRNAs in modifying mRNA translation and their potential involvement in human diseases were revealed (3, 4). We now know that miRNAs play an important role in central nervous system (CNS) development and function (5, 6) and that dysregulation of miRNAs is tied to alterations in behavior and cognition seen in a number of neuropsychiatric disorders (7). Here, we focus on miRNAs identified in studies of humans with autism spectrum disorder (ASD).

Brain miRNA expression was first examined in postmortem cerebellum from ASD subjects (8). A succession of ensuing studies reported widespread miRNA dysregulation in the CNS and periphery, including lymphoblasts (9–11), blood (12–14), saliva (15), and olfactory precursor cells (16). These studies have produced vast amounts of information, some unifying and some conflicting. The way in which reports of circulating miRNAs instruct our understanding of miRNAs in the CNS is still being explored (17). To guide that exploration, this review summarizes current knowledge of miRNA in ASD, with the goal of identifying the most consistent findings across studies with potential implications for biomarker discovery.

### Clinical Aspects of Autism Spectrum Disorder

Autism spectrum disorder is a heterogeneous disorder typified by deficits in social communication and restricted, repetitive patterns of behavior (18). Children with ASD are often adherent to daily routines, preoccupied with specific topics, and over sensitive to sensory inputs such as sounds or textures (19). In most cases, ASD symptoms are first recognized in early childhood. The average age of ASD diagnosis is 4 years (20). Rates of ASD are increasing. Recent reports from the Centers for Disease Control and Prevention estimate the prevalence of ASD to be 1:68 children (1:45 among males) (21). This increase may be the result of multiple factors including public awareness, changes in diagnostic criteria, and environmental influences. However, genetics also plays an important role in ASD (22).

### Genetic Contributions to ASD

A large number of familial studies demonstrate that ASD is a heritable disorder (23, 24), with estimated genetic contributions accounting for >50–60% of ASD risk (25). Indeed, a large number of genetic variants and chromosomal abnormalities are linked to ASD (26). These variants tend to be highly penetrant but rare. However, in some cases, common variants with low penetrance have been reported (27). Neither explains the incidence of ASD in the general population. Genetic epidemiologic studies have also shown that ASD is not a single disease but a constellation of symptoms involving multiple gene networks (28). Posttranscriptional mechanisms, such as miRNA, that broadly influence gene expression without altering the DNA code represent one means of altering entire gene networks (29). Increasingly, investigators have turned toward these mechanisms to explain the dysregulation of neurodevelopmental pathways that occurs in ASD (30, 31).

### miRNAs Regulate Posttranscriptional Gene Expression

MicroRNAs are short (18–25 nucleotide), non-coding RNA molecules (32) that influence gene expression and numerous cellular processes, including proliferation, differentiation, and apoptosis (33). Over 2500 mature miRNAs (and 1800 precursors) have been identified in humans (34). Many of these are evolutionarily conserved (35). The genes for miRNAs are found in inter- or intra-genic regions (36). They are transcribed into precursor hairpin structures (pri-miRNAs) by RNA polymerase-II and trimmed into pre-miRNA by the RNase Drosha. After leaving the nucleus, Dicer liberates the pre-miRNA into a single-stranded molecule that can be bound within the miRNA-induced silencing complex (miRISC) (37). The targeting of the mature miRISC to individual mRNAs is based on the “seed sequences” that reside at the 5′ end of the miRNA and are complementary to the 3′ regions of mRNAs. Each seed sequence aligns with hundreds of mRNAs, and there are several miRNAs with shared seed sequences (38). Once bound to a target mRNA, the miRISC complex reduces the efficiency of gene expression by repressing protein translation or promoting degradation of the mRNA transcript (37).

### miRNAs in the Brain and Periphery

MicroRNAs regulate approximately two-thirds of human mRNAs (39). Each miRNA has hundreds of potential mRNA targets, giving it the ability to modulate entire gene networks. In addition, miRNAs can circulate within exosomes, microvesicles, or RNA-binding proteins such as high-density lipoproteins, and thereby travel extracellularly to alter gene expression in distant tissues. This renders them exceedingly stable and easily measured in serum, plasma, saliva, urine, and other biofluids (40). However, miRNAs are most ubiquitous in the CNS, which expresses an estimated 70% of all miRNAs (41). Notably, expression of brain miRNAs changes throughout childhood and varies across brain regions (42). Neurons and glia are abundant sources of miRNAs, which can be readily transported across the blood–brain barrier (BBB). Moreover, miRNAs in neurons also help compartmentalize or localize mRNA expression and translation within specific subcellular regions such as axons and dendrites.

### The Role of miRNAs in Neurodevelopment

MicroRNAs play important roles in neurogenesis, synaptogenesis, and neuronal migration (43, 44). A major means by which these roles are manifest is the effect that neuronal miRNAs have on spatial localization or compartmentalization of protein translation in different neuronal subregions, such as axons, dendrites, and synapses (45, 46). Recent data support the relevance of these processes for neurodevelopmental disorders. For example, in mice with fragile X-associated tremor/ataxia syndrome (FXTAS), the fragile X mental retardation (FMR1) transcript is targeted by miR-221, miR-101, and miR-129-5p (47). Synaptosomal preparations from FXTAS mice demonstrate dysregulation of transcripts in learning and social interaction along with reduced levels of miR-221. Other examples include studies of disruption of miRNA regulatory genes such as Dicer or DGCR8, which produce alterations in synaptic plasticity within the prefrontal cortex (48). The gene for DGCR8 is located at 22q11.21 and a well-characterized hemi-deletion of this region (referred to as velocardiofacial syndrome) produces a complex phenotype along with ASD traits in approximately one-third of subjects (49). Moreover, mice with DGCR8 haploinsufficiency have reduced dendrite complexity, fewer neuronal spines, and decreased neurogenesis (50). Complementing these data, dysregulation of miRNA expression has been described in a number of developmental, neuropsychiatric, and neurodegenerative disorders including schizophrenia, Alzheimer’s disease, and Parkinson’s disease (36).

### The Role of miRNAs in ASD Pathogenesis

Four studies examining postmortem brain tissue of human subjects with ASD have identified 91 miRNAs with differential expression compared with typically developing controls. The exact pathophysiologic role of these miRNAs in ASD remains unclear; however, many are implicated in neurogenesis and synaptogenesis. A study of cerebellar cortex in 13 adults with ASD (8) identified 28 miRNAs with differential expression and 7 of these targeted the SHANK3 transcript. SHANK3 is involved in regulation of synaptic density and has copy number variants (CNVs) and point mutations in nearly 1% of ASD cases (51). A study by Wu et al. (52) examined cerebellar cortex in 28 ASD subjects. The authors found miR-21-3p was upregulated in ASD subjects and demonstrated that its overexpression in human neuroprogenitor cells could repress multiple M16 hub genes, including DLGAP1 (a scaffold protein that interacts with SHANK3). Thus, SHANK3 represents one pathophysiologic target with an established role in ASD that appears to be targeted by multiple miRNAs identified in multiple studies of ASD brain tissue.

### Overlapping miRNAs in ASD

This review synthesizes the findings of 12 studies, which compared miRNA expression in human cases of ASD to miRNA profiles in healthy controls (Table 1). Four of these studies involved brain tissue (8, 52–54), three involved peripheral blood (12–14), one examined saliva (15), one examined olfactory precursor cells (16), and three employed cultured lymphoblasts (9–11).

**Table 1:** **miRNA biomarker study characteristics and findings**.

	Abu-ElNeel et al. (8)	Mor et al. (53)	Ander et al. (54)	Wu et al. (52)	Vasu et al. (13)	Huang et al. (14)	Popov et al. (12)	Hicks et al. (15)	Nguyen et al. (16)	Sarachana et al. (10)	Talebizadeh et al. (9)	Seno et al. (11)
Biomaterial	Postmortem cerebellar cortex	Postmortem Brodmann’s area 10	Postmortem Brodmann’s areas 22, 41, and 42	Postmortem cerebellar cortex, Brodmann’s area 9	Serum	Peripheral blood	Whole blood	Saliva	Olfactory mucosal stem cells + skin fibroblasts or PBMCs	Lymphoblast cell lines	Lymphoblast cell lines	Lymphoblast cell lines

# participants	13 ASD, 13 control	12 ASD, 12 control	10 ASD, 8 control	28 ASD, 28 control	55 ASD, 55 control	5 ASD/5 control (microarray), 15ASD/15 control (qRT-PCR)	30 ASD, 25 control	24 ASD, 21 control	8 ASD, 6 control	14 ASD, 14 sibling controls (3 monozygotic twins)	6 ASD, 6 controls	20 ASD, 22 sibling controls

ASD characteristics	Not described	Age 30 years, 83% male, ADI-R (no scores provided), PMI 26 h	Age 31 years, 50% male, some ADI-R, Autism Tissue Program, PMI 23 h	Age 31 years, 82% male, some ADI-R, 17% with 15q duplication, PMI 24 h	Age 11 years, 87% male, ADI-R (35), Japanese	Age 5 years, 80% male, DSM-4 criteria, Chinese	Age 8 years, 80% male, DSM-4 criteria, Bulgarian	Age 9 years, 79% male, ADOS (10.6), Vineland Adaptive Behavior (71)	Age 30 years, 70% male, French	No ages, 100% male, Autism Genetics Resource Exchange	Age 10 years old, 50% male, ADI-R (no scores), Autism Genetics Resource Exchange	No age or sex, ADOS (“severe,” but no scores), European

Measurement technique	qRT-PCR	RNA-Seq with qRT-PCR validation	miRNA microarray, no validation	RNA-seq, qRT-PCR validation	PCR array with qRT-PCR validation	miRNA microarray with qRT-PCR validation	Microarray	RNA-seq	Microarray with qRT-PCR validation	miRNA microarray with qRT-PCR validation	miRNA microarray with qRT-PCR validation	miRNA microarray with qRT-PCR validation

# miRNAs tested	377	1104	1733	699	125	2578	Unknown	246	667	1237	150	708

# differentially expressed	28	20	6	58	14	44	1	14	4	43	9	16

% differentially expressed	7	2	0.3	8	11	2	Unknown	6	0.6	3	6	2

Criteria for differential expression	*z*-test of individual ASD Ct vs. pooled controls w/FDR	Two-tailed *t*-test with FDR < 0.05	*p* < 0.005, FC > 1.2	Linear mixed effects model, FDR < 0.05	Mann–Whitney pooled samples *p* < 0.05	Mann–Whitney *p* < 0.05	Welch’s *p* < 0.05, signal intensity >3	Mann–Whitney with FDR < 0.15	Mann–Whitney *p* < 0.05	Pavlidis Template Matching (PTM) analysis	*p* < 0.05 after Bonferroni, FC 1.5	1.5 FC in ≥12/24 sibling-pair comparisons

# overlapping with other studies	29	35	0	14	14	16	0	43	1	25	44	13

Largest change	miR-484 (downregulated in 3 ASD participants)	miR-338-5p (4.4 FC, *p* = 5.47E−81)	miR-297 (−1.24 FC, *p* = 0.0012)	miR-3687 (−1.5 FC, FDR 1.5E−4)	miR-572	miR-451a (*p* = 4.58E−5)	miR-486-3p (*p* = 0.03)	miR-628-5p (*Z* diff 1.13, FDR 0.027)	miR-146a (*p* < 0.001)	miR-182-AS (−1.54 FC, 1.44E−03)	miR-132 (changed in all 6 ASD individuals)	miR-199b-5p (1.81 FC, *p* = 2.51E−05)

*ADI-R, Autism Diagnostic Inventory – Revised; ADOS, Autism Diagnostic Observation Schedule; ASD, autism spectrum disorder; Ct, cycles-to-threshold; FC, fold change; FDR, false discovery rate correction; PMI, postmortem interval; qRT-PCR, quantitative reverse-transcription/real time polymerase chain reaction; RNA-Seq, ribonucleic acid sequencing; sib, sibling*.

The 12 studies together identified 219 miRNAs with potential implications in ASD (Table 2A), including 185 unique and 34 overlapping miRNAs (15.5%). Twenty-seven overlapping miRNAs changed in the same direction (Table 2B) in ≥2 studies, and 3 changed in the same direction in ≥3 studies (miR-23a, miR-146a, and miR-106b). Notably, 14 of the 27 miRNAs shared seed sequences with other ASD-associated miRNAs, suggesting that the pathophysiology of miRNA dysregulation in ASD might be controlled, at least in part, at the seed level. There is no single miRNA identified in all the 12 studies. This may be a function of ASD heterogeneity, miRNA tissue specificity, or advances in RNA quantification, which have led to varying alignment techniques and identification of new miRNAs.

**Table 2:** **Cross-tissue miRNA biomarkers with seed sequences**.

A.	Brain				Serum			Saliva	Lymphoblast			Olfactory SCs

	Abu-ElNeel et al. (8)	Mor et al. (53)	Ander et al. (54)	Wu et al. (52)	Vasu et al. (13)	Huang et al. (14)	Popov et al. (12)	Hicks et al. (15)	Sarachana et al. (10)	Talebizadeh et al. (9)	Seno et al. (11)	Nguyen et al. (16)
Up-regulated	miR-106a	**miR-7-5p**	miR-664-3p	**miR-10a-5p**	**miR-19b-3p**	miR-34b-3p		**miR-7-5p**	miR-16-2	miR-23a	**miR-10a**	**miR-146a**
	**miR-106b**	miR-19a-3p	miR-4709-3p	miR-18b-5p	miR-27a-3p	miR-34c-3p		miR-28-5p	**miR-106b**	miR-23b	miR-30a	
	**miR-140**	**miR-19b-3p**	miR-4753-5p	miR-20b-5p	miR-101-3p	miR-483-5p		miR-127-3p	**miR-132**	**miR-132**	miR-181a	
	**miR-146b**	**miR-21-3p**		**miR-21-3p**	**miR-106-5p**	**miR-494**		**miR-140-3p**	miR-133b	**miR-146a**	miR-181b	
	miR-181d	miR-21-5p		**miR-23a-3p**	miR-130a-3p	miR-564		miR-191-5p	miR-136	**miR-146b**	miR-181c	
	miR-193b	miR-142-3p		miR-107	miR-195b-5p	miR-574-5p		miR-218-5p	miR-139	miR-663	**miR-199b-5p**	
	miR-320a	miR-142-5p		miR-129-2-3p		miR-575		**miR-335-3p**	miR-148b		***miR-338-3p***	
	miR-381	miR-144-3p		miR-130b-5p		miR-921		miR-628-5p	miR-153		miR-486-3p	
	miR-432	**miR-146a-5p**		miR-148a-3p		miR-1246		miR-2467-5p	miR-182		miR-486-5p	
	miR-539	**miR-155-5p**		**miR-155-5p**		miR-1249		miR-3529-3p	miR-189		miR-500	
	miR-550	**miR-219-5p**		miR-218-2-3p		miR-1273c			miR-190		miR-502-3p	
	miR-652	**miR-338-5p**		miR-221-3p		miR-4270			**miR-199b**		miR-548	
		miR-379-5p		miR-223-3p		miR-4299			miR-211			
		miR-451a		**miR-335-3p**		miR-4436a			**miR-219**			
		**miR-494**		miR-363-3p		miR-4443			miR-326			
		miR-3168		miR-424-3p		miR-4516			miR-367			
				miR-424-5p		miR-4669			miR-455			
				miR-425-3p		miR-4721			miR-495			
				miR-449b-5p		miR-4728-5p			miR-518a			
				miR-450b-5p		miR-4788			miR-520b			
				miR-484		miR-5739						
				miR-629-5p		miR-6086						
				miR-651-5p		miR-6125						
				miR-708-5p		miR-642a-3p						
				miR-766-3p								
				miR-874-3p								
				miR-887-3p								
				miR-940								
				miR-1277-3p								
				miR-3938								
				miR-2277-5p								
				let-7g-3p								

Down-regulated	miR-7	miR-34a-5p	miR-1	miR-204-3p	miR-151a-3p	**miR-15a-5p**	miR-486-3p	**miR-23a-3p**	**miR-23a**	**miR-92**	*miR-199a-5p*	miR-221
	**miR-15a**	**miR-92b-3p**	miR-297	miR-491-5p	miR-181b-5p	**miR-15b-5p**		**miR-27a-3p**	miR-23b	**miR-320**	*miR-455-3p*	miR-654-5p
	**miR-15b**	miR-211-5p	miR-4742-3p	miR-619-5p	**miR-320a**	miR-16-5p		**miR-30e-5p**	miR-25	miR-363	*miR-577*	miR-656
	miR-21	miR-3960		miR-3687	miR-328	miR-19b-3p		miR-32-5p	miR-29b		*miR-650*	
	**miR-23a**			miR-5096	miR-433	miR-20a-5p			**miR-30e**			
	**miR-27a**				miR-489	**miR-92a-3p**			**miR-93**			
	***miR-93***				miR-572	**miR-103a-3p**			**miR-103**			
	miR-95				miR-663a	**miR-195-5p**			miR-107			
	miR-128					**miR-451a**			miR-185			
	miR-129					miR-574-3p			miR-186			
	miR-132					miR-940			miR-191			
	miR-148b					miR-1228-3p			miR-194			
	miR-212					miR-3613-3p			**miR-195**			
	miR-431					miR-3935			miR-205			
	miR-484					miR-4436b-5p			miR-342			
	miR-598					miR-4665-5p			miR-346			
						miR-4700-3p			miR-376a-AS			
						let-7a-5p			**miR-451**			
						let-7d-5p			miR-519c			
						let-7f-5p			miR-524			

								**Direction of change in different biomaterials**				
**B.**	**miRNA ID**	**Seed sequence**	**Human miRNAs with same seed**					**CNS**	**Blood**	**Saliva**	**Lymphoblast**	**Olfactory**

	miR-7-5p	GGAAGAC						↑↓		↑		
	miR-10a-5p	ACCCUGU	miR-10a-5p miR-10b-5p					↑			↑	
	miR-15a-5p	AGCAGCA	miR-15a-5p miR-15b-5p **miR-16-5p miR-195-5p** miR-424-5p miR-497-5p [miR-6838-5p]					↓	↓			
	miR-15b-5p	AGCAGCA	miR-15a-5p miR-15b-5p **miR-16-5p miR-195-5p** miR-424-5p miR-497-5p [miR-6838-5p]					↓	↓			
	miR-19b-3p	GUGCAAA	**miR-19a-3p**					↑	↑↓			
	miR-21-3p	AACACCA	miR-3591-3p					↑↑				
	miR-23a-3p	UCACAUU	miR-23-3p **miR-23b-3p** miR-23c [miR-130a-5p]					↑↓		↓	↓↑	
	miR-27a-3p	UCACAGU	miR-27-3p miR-27b-3p					↓	↑	↓		
	miR-30e-5p	GUAAACA	*miR-30a-5p* miR-30b-5p miR-30c-5p miR-30d-5p miR-30-5p							↓	↓↑	
	miR-92a-3p	AUUGCAC	**miR-25-3p miR-32-5p** miR-92-3p miR-92b-3p **miR-363-3p** *miR-367-3p*						↓		↓	
	miR-92b-3p	AUUGCAC	**miR-25-3p miR-32-5p** miR-92-3p miR-92b-3p **miR-363-3p** *miR-367-3p*					↓			↓	
	miR-93	AAAGUGC	miR-17-5p **miR-20a-5p** miR-20b-5p miR-93-5p *miR-106a-5p miR-106b-5p* miR-519d-3p miR-526b-3p					↓			↓	
	miR-103a-3p	GCAGCAU	miR-103-3p						↓		↓	
	miR-106b-5p	AAAGUGC	miR-17-5p *miR-20a-5p* miR-20b-5p miR-93-5p **miR-106a-5p** miR-519d-3p [miR-526b-3p]					↑	↑		↑	
	miR-132	AACAGUC	miR-132-3p *miR-212-3p*								↑↑	
		CCGUGGC	miR-132-5p									
	miR-140-3p	ACCACAG						↑		↑		
	miR-146a-5p	GAGAACU	miR-146a-5p **miR-146b-5p** [miR-7153-5p]					↑			↑	↑
	miR-146b	GAGAACU	**miR-146a-5p miR-146b-5p** [miR-7153-5p]					↑			↑	
	miR-155-5p	UAAUGCU						↑↑				
	miR-195-5p	AGCAGCA	**miR-15a-5p miR-15b-5p miR-16-5p** miR-424-5p miR-497-5p [miR-6838-5p]						↓↑		↓	
	miR-199b-5p	CCAGUGU	*miR-199a-5p*								↑↑	
	miR-219-5p	GAUUGUC	miR-219a-5p [miR-4782-3p] [miR-6766-3p]					↑			↑	
	miR-320a	AAAGCUG	miR-320b miR-320c miR-320d [miR-4429]					↑	↓		↓	
	miR-335-3p	UUUUCAU						↑		↑		
	miR-338-5p	ACAAUAU						↑			↑	
	miR-451a	AACCGUU						↑	↓		↓	
	miR-494	GAAACAU	miR-494-3p					↑	↑			
		GGUUGUC	miR-494-5p miR-323b-5p miR-410-5p									
			% overlap					23.1	19.0	42.8	25.8	25.0

*Boldface miRNAs change in the same direction in multiple tissues. Italic miRNAs change in different directions in multiple studies. Lower left indicates miRNAs that share the same seed sequence as those that appear changed in multiple studies. Lower right indicates predominant direction of change in ASD subjects relative to controls for each biomaterial. For tissues with opposing changes both arrows are shown*.

*CNS, central nervous system; SCs, stem cells*.

*The final row shows the proportion of altered miRNAs for each biomaterial that overlap with other studies*.

### Functional Significance of Overlapping miRNAs and Their Gene Targets in ASD

Of the 27 overlapping miRNAs, 22 show differential regulation in studies of the CNS. The remaining five are expressed in the developing brain (42, 53). These 27 were predicted to target 1245 mRNAs (Table S1 in Supplementary Material) with target scores ≥93 (55).^1^ Notably, 86 of these mRNAs overlapped with the 519 human ASD candidate genes with supportive evidence existing in the Simons Foundation Autism Research Initiative (SFARI) database,^2^ representing a 6.6-fold enrichment for ASD-associated genes compared to chance (odds ratio: 7.7, 95th CI = 6.0–9.9, *z* = 16.2, *p* < 0.0001).

We next examined the evidence for functional enrichment of specific biologic processes using the Database for Annotation, Visualization, and Integrated Discovery (DAVID). This revealed 225 total cluster mappings (56), with the top nodes including a KEGG pathway involved in neurotrophin signaling [*n* = 25 genes, false discovery rate (FDR) = 0.0018]. In fact, this node had the largest fold change (FC) (3.0-fold) among all detected clusters (Table S2 in Supplementary Material). DAVID enrichment analysis also identified 662 (55.9%) of the target mRNAs as expressed in brain tissue, with additional enrichment of genes expressed in epithelium (*n* = 268), fetal kidney (*n* = 35), placenta (*n* = 275), amygdala (*n* = 61), and T-cells (*n* = 37) (Table S3 in Supplementary Material).

To further examine functional relevance of the most probable mRNA targets, the Ingenuity Pathway Analysis (IPA) databases for “Canonical Pathways” and “Diseases and Biological Function” were used. These analyses were performed using a restricted set of 293 mRNAs that were predicted to be targets of the 27 overlapping miRNAs with miRDB target scores ≥95. The results indicated enrichment of 20 Canonical Pathways (FDR < 0.05), including two related to neurotrophin signaling (Table S4 in Supplementary Material). Moreover, the “Diseases and Biological Function” analysis yielded a list of 500 networks for the 293 mRNA targets, including 93 (18.6%) involved in brain function, of which 35 survived FDR correction (Table S5 in Supplementary Material). These represented several functions, including brain development, neurogenesis, neural and glial differentiation, synaptic development and function, and cognitive and motor function. The diseases identified among the brain-related networks included schizophrenia, abnormal brain morphology and trigeminal nerve morphology, abnormal posture, cognitive impairment, and seizure disorders.

Upstream Regulatory analysis was performed in IPA considering only experimentally observed and high-confidence miRNA–mRNA interactions, which reduced our list of 27 miRNAs to 20. Notably, 199 mRNAs were targeted by ≥2 miRNAs, and 55 genes were targeted by ≥5 of the 20 miRNAs. The significance of this was determined using a Fisher’s exact test, which confirmed enrichment for 49 mRNAs (Table S6 in Supplementary Material). Protein–protein interaction (PPI) networks were also explored in the 199 target genes using the STRINGv10 database,^3^ which yielded a significant PPI network containing 137 edges and several nodes involved in brain function (Figure S1 in Supplementary Material). Among this network was an intriguing interaction between neuronal cell adhesion molecule (NRCAM; involved in directional signaling of axonal cone growth and implicated in ASD) (57), semaphorin 3a (SEMA3A; a chemoattractive agent involved in axon guidance) (58), and brain-derived neurotrophic factor (BDNF; essential for synaptogenesis in the developing hippocampus and altered in the periphery of ASD subjects) (59).

### Expression of miRNA across Brain Regions

MicroRNA expression is a dynamic process, changing throughout development and across brain regions (43). A study of non-coding RNA in the superior temporal gyrus of ASD subjects demonstrated changes in regional miRNA profiles that could contribute to ASD (60). Specifically, while miRNA patterns in the superior temporal sulcus (a region relevant to social interaction) and primary auditory cortex changed throughout development in the typical brain, many of the changes were blunted in subjects with ASD. Four of the individual miRNAs identified (miR-93-3p, miR-103, miR-132, and miR-320) were among the 219 miRNAs altered in other studies of ASD, with 3 of the 4 changed in multiple studies (Table 2).

### Potential Mechanisms for miRNA Dysregulation

The molecular mechanisms underlying miRNA dysregulation in ASD are still being explored. One explanation is that autistic miRNA patterns may represent a permanent response to an early environmental insult, such as perinatal hypoxia (61). In this scenario, miRNA regulation could be disrupted by a layer of epigenetic machinery such as DNA methylation. In a study of 12 subjects with ASD, expression of miR-142 was increased in Brodmann’s area 10 compared with healthy controls – a change accompanied by hypomethylation of the miR-142 promoter region (53).

Another possible mechanism is that individual miRNAs sequences are altered in children with ASD. Pooled analysis of common and rare single nucleotide polymorphisms (SNPs) in 449 cases of ASD using whole exome sequencing has identified 2 miRNA clusters: miR-133b/miR-206 and mir-17/miR/18a/miR-19a/miR20a/miR-19b-1/miR-92a (62). Four of these individual components have disrupted expression levels in children with ASD, and miR-92a is downregulated in multiple studies (9, 14).

Yet another explanation could be that the location of specific miRNAs at CNV loci leads to their dysregulation. Of the 41 CNV loci associated with ASD, >10% contain miRNAs (63). Three of the 10 “hub miRNAs” (i.e., miRNAs at CNV loci with the greatest gene network connectivity) have been previously identified in studies of ASD (miR-34a, miR-548, and miR-195) and target multiple genes involved in neurodevelopment. Alterations in miR-195 have been reported in serum (13, 14) and lymphoblasts (10) of ASD individuals. Furthermore, a key gene involved in miRNA synthesis (Dicer) was identified as a common target for many of the hub miRNAs, suggesting that some CNVs in ASD might cause extensive dysregulation of the entire miRNA machinery.

### Systemic miRNA Dysregulation

Although the brain is considered the primary pathological target in ASD, alterations in miRNA expression have been noted in a number of other body sites (13, 15). This may explain (in part) why children with ASD experience medical comorbidities outside the CNS, including gastrointestinal issues such as esophageal reflux and constipation (64). They also have altered oral-motor function (speech apraxia) and sensory processing (65, 66). Given these comorbidities, it should come as no surprise that oral miRNA patterns are altered in children with ASD. In addition, the proximity of the oropharynx to the CNS and its abundant sensory and motor nerve innervation (involving branches of the glossopharyngeal, facial, vagus, and trigeminal nerves) could provide an ample source of miRNA (67, 68).

Alterations in miRNA profiles of peripheral blood sites may be explained by circulating miRNA that has crossed the BBB (especially considering that ASD is not associated with hematologic disorders). In peripheral sources such as blood, brain-related miRNA may be stable and abundant as a result of exosomal transport (13, 14). Thus, while biofluids such as serum and saliva may not represent optimal models for studying CNS pathophysiology, they may represent ideal reservoirs for ASD biomarkers.

### Employing miRNAs as Biomarkers

The potential of miRNAs as biomarkers in ASD has been explored in studies of serum and saliva. In a study of serum miRNAs from 55 children with ASD, the authors identified three miRNAs (miR-130-3p, miR-181b-5p, and miR-320a) with an area under the curve (AUC) >0.85 (13). In our own more recent study, involving 24 children with ASD, a set of 14 salivary miRNAs showed more than 95% accuracy (AUC = 0.92) at differentiating control and ASD subjects (15). This study also showed that expression patterns of individual salivary miRNAs were significantly correlated with several measures of adaptive behavior. This highlights how the utility of miRNA extends beyond simple ASD diagnosis and may 1 day be used to predict ASD phenotype and severity.

## Conclusion

It is clear that miRNA profiles are dysregulated across multiple tissue types in subjects with ASD. This review encapsulated 219 target miRNAs from 12 human studies of ASD and identified 27 that were dysregulated in ≥2 investigations. Functional pathway analysis supports the idea that these miRNAs target brain-expressed genes related to neurodevelopment and implicated in ASD. Three miRNAs showed consistent dysregulation across ≥3 studies (miR-23a-3p, miR-146a-5p, and miR-106b-5p). The networks of genes targeted by these miRNAs are implicated in ASD and have significant roles in neurotrophin signaling. BDNF, which is important for hippocampal neurogenesis and decreased in the serum of adult ASD patients (59), is targeted by 6 of the 27 miRNAs of interest. Animal and cell models that assess these molecular mechanisms behind miRNAs and their mRNA targets will be critical in advancing the current ASD knowledge-base.

## Ethics Approval and Consent

This manuscript involves a review and analysis of current literature collected in preparation for the study “Improving Autism Screening with Brain-Related miRNA,” which has been approved by the Institutional Review Board at the Penn State College of Medicine, Hershey, PA, USA (STUDY00003658). According to the publications on which this review was based, informed consent or assent was obtained from all participants or their parents, unless the study involved exclusively postmortem human brain material. In the case of minor children unable to provide informed assent, parental consent was obtained.

## Author Contributions

SH and FM conceived of this study, prepared the figures and tables, and wrote the manuscript.

## Conflict of Interest Statement

SH and FM hold a provisional patent for the use of a panel of specific miRNAs in the saliva as biomarkers for autism. The patent was filed by the State University of New York, Upstate Medical University, and has been licensed to Motion Intelligence, Inc. SH also receives consulting fees from Motion Intelligence, Inc. These interests have been disclosed and are under active management by the Penn State Conflict of Interest Committee.

## Footnotes

^1^http://mirdb.org.

^2^https://gene.sfari.org/autdb/HG_Home.do.

^3^http://string-db.org/.

## Supplementary Material

Figure S1 **Protein–protein interaction (PPI) network of the genes that are targeted by 2 or more of at least 20 of the 27 conserved miRNAs affected across studies**. This analysis involved mapping of 199 mRNA targets in the STRING v10 database (http://string-db.org/). Note the presence of a significant interaction network containing 137 edges for the 197 targets with annotation information available. Notably, only genes with connections are included in the PPI. Also, note that many of these genes were involved in brain-related functions, including some that mapped to a neuronal projection Gene Ontology (highlighted in red).

## Abbreviations

ADI-R, autism diagnostic interview – revised; ADOS, Autism Diagnostic Observation Schedule; ASD, autism spectrum disorder; AUC, area under the curve; BBB, blood–brain barrier; CNS, central nervous system; CNV, copy number variant; DAVID, Database for Annotation, Visualization, and Integrated Discovery; HDL, high-density lipoprotein; MeCP2, methyl CpG-binding protein; miRNA, microRNA; PPI, protein–protein interaction; SFARI, Simons Foundation Autism Research Initiative; SNP, single nucleotide polymorphism.
