# Supplementary figures and images for: A Comparative Review of microRNA Expression Patterns in Autism Spectrum Disorder
Source: Front Psychiatry. 2016 Nov 4;7:176. doi: 10.3389/fpsyt.2016.00176 (PMC5095455; doi:10.3389/fpsyt.2016.00176)

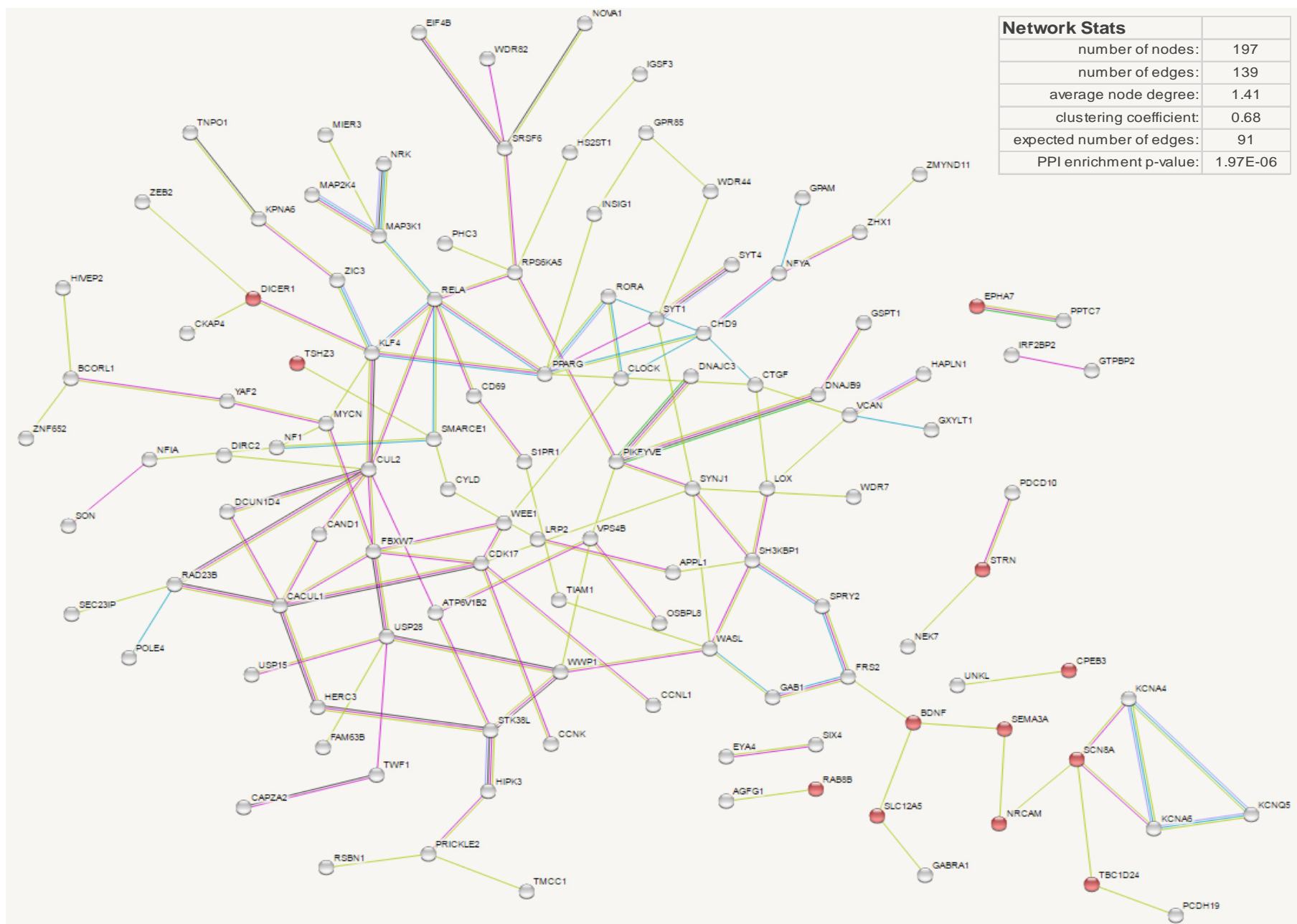

Supplement: Figure S1 — Protein–protein interaction (PPI) network of the genes that are targeted by 2 or more of at least 20 of the 27 conserved miRNAs affected across studies. This analysis involved mapping of 199 mRNA targets in the STRING v10 database (http://string-db.org/). Note the presence of a significant interaction network containing 137 edges for the 197 targets with annotation information available. Notably, only genes with connections are included in the PPI. Also, note that many of these genes were involved in brain-related functions, including some that mapped to a neuronal projection Gene Ontology (highlighted in red). [file Image_1.PDF]
